# FACTORS INFLUENCING COVID-19 VACCINE DECISION: WHAT ATTITUDES CAN WE EXPECT FROM YOUNG POLES IN THE FUTURE? A CROSS-SECTIONAL, REPRESENTATIVE SURVEY

**DOI:** 10.13075/ijomeh.1896.02595

**Published:** 2025

**Authors:** Aneta Tomaszewska, Barbara Bałan, Karolina Sobeczek, Kamil Rakocy, Konrad Furmańczyk, Mariola Chrzanowska, Piotr Samel-Kowalik, Filip Raciborski, Bolesław Samoliński

**Affiliations:** 1 Medical University of Warsaw, Department of Prevention of Environmental Hazards, Allergology and Immunology, Warsaw, Poland; 2 Medical University of Warsaw, Doctoral School, Warsaw, Poland; 3 KR Consulting, Warsaw, Poland; 4 Warsaw University of Life Sciences – SGGW, Department of Applied Mathematics, Warsaw, Poland; 5 Warsaw University of Life Sciences – SGGW, Department of Econometrics and Statistics, Warsaw, Poland

**Keywords:** Poland, cross-sectional study, vaccine hesitancy, vaccine refusal, medical misinformation, COVID-19 vaccination

## Abstract

**Objectives:**

This study aimed to identify factors influencing COVID-19 vaccination decisions and reasons for vaccine refusal among young Poles – a population with the lowest uptake in the country.

**Material and Methods:**

A nationwide cross-sectional study was conducted using the computer-assisted personal interview method on a representative sample of 1560 individuals aged 15–39 years. The multivariate logistic regression model was used to analyze the relationship between selected factors and COVID-19 vaccination status.

**Results:**

The likelihood of vaccination was significantly higher among women (odds ratio [OR] = 1.64), individuals aged 25–39 years (OR = 2.47), those with higher education (OR = 4.84), married (OR = 2.18), parents (OR = 2.35) and deeply religious respondents (OR = 4.97). The strongest predictor was fear of COVID-19 infection (OR = 28.14). Among vaccine-hesitant individuals, the most common concerns were vaccine safety (40%) and efficacy (35%). Others perceived COVID-19 as a mild illness (27%), believed prior infection provided sufficient immunity (22%), or preferred natural methods (14%). Vaccination status correlated with attitudes toward vaccines and the pandemic. The strongest positive correlations were with beliefs that vaccination protects others (r_s_ = 0.59), COVID-19 vaccines are a medical success (r_s_ = 0.51), and that experts promoting vaccines are credible (r_s_ = 0.45). Negative correlations were linked to misinformation, such as claims about genetic effects, unethical experimentation, or dangerous ingredients. The reasons for refusing the COVID-19 vaccine cluster into 2 groups: modifiable and non-modifiable. This division assumes that it is possible to intervene to modify some factors, while others are beyond our control.

**Conclusions:**

Vaccine hesitancy is shaped not only by lack of knowledge but also by mistrust and social polarization. Therefore, public health strategies should combine educational efforts with communication delivered through trusted channels. Otherwise, polarization may persist – with only part of the hesitant group open to change.

## Highlights

- Vaccine refusal is linked to misinformation and distrust in experts.
- Educating young Poles is key to improving vaccination rates.
- The main reasons for hesitation are safety, efficacy, low fear of COVID-19.

## INTRODUCTION

Although coronaviruses have been present in human history for many years, primarily causing diseases in animals or mild cold-like infections in humans, the mutation that emerged at the end of 2019 proved to be highly dangerous to health and life. It was the first instance in over a century of such a severe, deadly, and rapidly spreading infection, leading to a world-altering pandemic.

The entire situation was further complicated by the fact that the virus was highly contagious during the asymptomatic period (rapid spread from person to person, rapid virus mutation) and caused a previously unknown respiratory disease with a very severe, even fatal course, accompanied by numerous complications in other systems and organs, such as the central and peripheral nervous systems (loss of smell, taste), the heart and circulatory system (myocarditis, blood pressure fluctuations). It also caused hematological problems (thrombotic and lymphoproliferative syndromes) and diabetic issues (unstable blood sugar levels, diabetes) [1].

Initially, knowledge about the SARS-CoV-2 virus itself, the causal treatment of the disease (coronavirus disease 2019 – COVID-19) it caused, recommendations for procedures, and consistent media messaging on what the virus is, what the disease is, and how to protect oneself was limited. This coincided with societal concerns about immunization, manifested in the decline in mandatory vaccinations and the activities of anti-vaccine movements.

Vaccinations under the National COVID-19 Vaccination Program began in Poland in December 2020, initially targeting particularly vulnerable groups, such as healthcare workers, and then expanding the list of target groups. In May/June 2021, COVID-19 vaccinations became available to everyone >15 years old [2].

In Poland, vector vaccines and mRNA-based vaccines were available, which was new to the public and could raise additional concerns. Among the mRNA vaccines, the Pfizer-BioNTech vaccine Comirnaty and the Moderna vaccine (currently known as Spikevax) were used, with an efficacy of 94–95%. Additionally, the Oxford-AstraZeneca vector vaccine was used, with an estimated efficacy of 60–80% [3].

This study addresses a gap in current research, as no previous representative, nationwide study had comprehensively examined the attitudes of young people in Poland (aged 15–39 years) toward COVID-19 vaccination. Given that this age group demonstrated the lowest vaccination rates at the national level, the findings provide valuable insight into the behavioral and psychosocial factors driving vaccine hesitancy and acceptance.

The aim of the study was to identify the factors influencing the decision to get vaccinated against COVID-19 and the reasons for refusal among the young Polish population.

## MATERIAL AND METHODS

### Study design

A nationwide cross-sectional study was conducted on a representative sample of 1560 residents of Poland aged 15–39 years, of both genders. The study was carried out using the computer assisted personal interview (CAPI) technique in October 7 – November 28, 2021. During the 30-minute in-person interviews, trained interviewers read each question aloud to participants and entered their responses directly into the system. Participants did not fill in the questionnaire independently.

### The sample selection and implementation method

The study used a hybrid representative sample, combining address-based sampling and a random-route approach. A total of 1560 starting addresses were randomly selected based on the TERYT database (official register of Poland's territorial division). The sample consisted of 260 clusters, with each cluster containing 6 elements. A cluster represented a census district. Within each cluster, an interviewer conducted 6 interviews with respondents who met the study's participation criteria. The demographic structure (gender and age) of respondents was assigned to each cluster. Interviewers received information about individuals with whom they should conduct interviews within their assigned clusters. Addresses were selected to proportionally reflect the structure of voivodeships (provinces) and the size of localities. The study was conducted using an address-based sample, where the mechanism for replacing addresses followed the random route method.

Quality control was conducted on 25% of the completed interviews, in accordance with the standards of the European Society for Opinion and Market Research (ESOMAR) and the Interviewer Quality Control Program (Program Kontroli Jakości Pracy Ankieterów – PKJPA). The interviews lasted an average of 30 min.

The inclusion criteria comprised residency on the territory of Poland and being 15–39 years old for both genders. The exclusion criteria comprised lack of communicative command of Polish, major disability, or anticipated non-compliance with the protocol.

### Research tool

A proprietary questionnaire was used, containing 58 questions in total. Thirteen substantive questions and 6 demographic questions were adapted from surveys conducted by the Center for Public Opinion Research (Centrum Badania Opinii Społecznej – CBOS) (permission was obtained for their use). The questions covered opinions on vaccinations, as well as the respondent's decisions and experiences in this regard.

### Statistical methods

Descriptive statistics and contingency tables were used. The level of significance of differences was determined by means of the χ^2^ test. Results were considered statistically significant at p < 0.05, with a 95% confidence interval (CI). The multivariate logistic regression model was used to analyze the relationship between selected factors and COVID-19 vaccination status. All analyses were performed with R package.

### Correlation coefficient

The participants indicated their agreement with statements regarding preventive vaccinations, COVID-19 vaccinations, and the COVID-19 pandemic. A total of 21 statements were analyzed using a 5-point scale ranging from “strongly agree” to “strongly disagree.” Each response was assigned a rank, followed by calculating the Spearman's rank correlation coefficient between each statement and the declaration of being vaccinated against COVID-19. The results of the correlation coefficient range from 1 to –1, where 1 indicates absolute support for the statement, and –1 indicates absolute disagreement/disapproval of the statement. Interpretation of results: <0.3 – weak positive correlation, 0.4–0.6 – moderate positive correlation, 0.7–0.9 – strong positive correlation, 1 – perfect correlation; similar interpretations apply to negative values.

### Ethics

The study was acknowledged by the Bioethical Review Board at the Medical University of Warsaw (AKBE/134/2021). Consent for the study was given orally. Each study participant received verbal and written information about the purpose of the study, the implementer, source of funding, protection of personal data, and the possibility to withdraw from the study at any stage. Information about the personal data administrator and contact details in case of questions or concerns were also included.

## RESULTS

### Characteristics of the study sample

During the study, attempts were made to contact 11 405 addresses. A total of 1560 complete interviews were conducted. Based on the collected data, including characteristics of refusals, indicators monitoring the degree of sample execution were estimated. The cooperation rate was 20.9%, while the contact rate was 84.7%.

The study group consisted of 52.8% women, with a statistically significant age difference between genders (p < 0.05). More than half of the participants (57.4%) lived in urban areas, with 10.8% residing in cities with a population of >500 000. The majority of the group (53.2%) had secondary education, and 69.2% reported having a full-time job, while 7.4% reported having part-time or occasional work. About half of the participants (51.0%) were married, and 38.5% had children. The majority of the group (70.9%) identified as religious, with 4.4% considering themselves deeply religious. A more detailed breakdown of the characteristics of the study group can be found in the Table 1.

**Table 1:** Structure of the nationally representative sample, Poland, October 7 – November 28, 2021

Variable	Participants (N = 1560)	
	n	%
Gender		
male	737	47.2
female	823	52.8
Age		
15–19 years	209	13.4
20–24 years	302	19.4
25–29 years	230	14.7
30–34 years	310	19.9
35–39 years	509	32.6
Place of residence		
rural area	666	42.7
town		
<20 000 population	192	12.3
20 000–50 000 population	168	10.8
50 000–100 000 population	126	8.1
100 000–500 000 population	240	15.4
>500 000 population	168	10.8
Education		
primary/junior secondary	183	11.8
secondary vocational	253	16.3
general/technical secondary with final examination passed	823	53.2
higher (bachelor's/master's degree)	289	18.7
Employment status		
yes		
full-time	1073	69.2
part-time or occasionally	114	7.4
no	364	23.5
Marital status		
unmarried	789	51
married	722	46.6
divorced/in separation/widowed	37	2.4
Children		
yes	600	38.5
no	960	61.5
Religiosity (self-declared)		
completely non-religious	103	6.6
rather non-religious	179	11.5
religious	1106	70.9
profoundly religious	68	4.4
refused to answer	104	6.7

### COVID-19 vaccination status

According to participants' declarations, 52% of the respondents have been vaccinated against COVID-19, with the majority being women and individuals >29 years of age. Fourteen percent of the respondents indicated they plan to get vaccinated, while 33% stated they do not intend to get vaccinated at all. In this group, men and individuals <25 years of age predominated. Detailed breakdown by gender and age groups is presented in Table 2.

**Table 2:** COVID-19 vaccination status among a nationally representative sample, Poland, October 7 – November 28, 2021

Variable	Participants (N = 1536^a^) [%]		
	vaccinated (N = 811, 52%)	not vaccinated (N = 725, 47%)	
		intending to get vaccinated (N = 218, 14%)	not intending to get vaccinated (N = 507, 33%)
Gender			
male	45	14	39
female	58	14	27
Age			
15–19 years	30	25	44
20–24 years	42	13	44
25–29 years	49	17	33
30–34 years	59	13	25
35–39 years	63	10	26

Frequency of answers to “Have you been vaccinated against COVID-19?” in total, by gender and age group.

a Non-response was observed in 24 cases out of 1560.

### Demographic and socio-economic factors related with COVID-19 vaccination

The decision to get vaccinated is significantly influenced by female gender (OR = 1.64, 95% CI: 1.34–2.00) and age 25–39 years (OR = 2.47, 95% CI: 1.98–3.07, ref.: 15–24 years). The size of respondents' place of residence is not statistically significant. Education level of respondents is significant; higher education increases the chance of vaccination (higher: OR = 4.84, 95% CI: 3.20–12.79, ref.: primary). Marital status of respondents also matters (married: OR = 2.18, 95% CI: 1.97–2.39, ref.: single) as well as having children (OR = 2.35, 95% CI: 2.14–2.56, ref.: no children). The likelihood is several times higher among individuals identifying as deeply religious (OR = 4.97, 95% CI: 4.31–5.63, ref.: total unbeliever) and those significantly afraid of coronavirus infection (OR = 28.14, 95% CI: 15.77–50.20, ref.: definitely not afraid) (Table 3).

**Table 3:** A multivariate logistic regression model for influence of selected factors on support for COVID-19 vaccination among a nationally representative sample, Poland, October 7 – November 28, 2021

Variable	Participants (N = 1536^a^)				OR	95% CI	p
	vaccinated (N = 811)		unvaccinated (N = 725)				
	%	n	%	n			
Gender							
male (ref.)	46.33	335	53.67	388			
female	58.55	476	41.45	337	1.64	1.34–2.00	<0.001
Age							
15–24 years (ref.)	37.90	191	62.10	313			
25–39 years	60.08	620	39.92	412	2.47	1.98–3.07	<0.001
Place of residence							
rural area (ref.)	50.00	331	50.00	331			
town							
<20 000 inhabitants	59.78	110	40.22	74	1.49	1.15–1.82	0.169
20 000–50 000 inhabitants	42.68	70	57.32	94	0.75	0.40–1.09	0.176
50 000–100 000 inhabitants	56.35	71	43.65	55	1.25	0.95–1.55	0.196
100 000–500 000 inhabitants	55.51	131	44.49	105	1.30	0.91–1.67	0.152
>500 000 inhabitants	59.76	98	40.24	66	1.48	1.14–1.83	0.177
Education							
primary (ref.)	25.28	45	74.72	133			
vocational	47.98	119	52.02	129	2.73	1.79–9.48	<0.05
secondary	57.16	463	42.84	347	3.94	2.74–11.25	<0.05
higher	62.11	177	37.89	108	4.84	3.20–12.79	<0.05
Marital status							
single (ref.)	43.39	338	56.61	441			
married	62.55	446	37.45	267	2.18	1.97–2.39	<0.001
divorced, separated	64.71	22	35.29	12	2.39	1.67–3.11	<0.05
Children							
no (ref.)	44.83	425	55.17	523			
yes	65.65	386	34.35	202	2.35	2.14–2.56	<0.001
Religiosity							
total unbeliever (ref.)	31.07	32	68.93	71			
unbeliever	44.89	79	55.11	97	1.81	1.30–2.33	<0.05
believer	56.35	617	43.65	478	1.25	0.82–1.68	<0.001
deep believer	69.12	47	30.88	21	4.97	4.31–5.63	<0.001
Employment status							
not working (ref.)	37.64	134	62.36	222			
part-time work	39.82	45	60.18	68	1.10	0.71–1.69	>0.05
full-time work	59.19	628	40.81	433	2.40	1.88–3.08	<0.05
Fear of coronavirus infection							
definitely no (ref.)	22.26	61	77.74	213			
no	38.78	190	61.22	300	2.21	1.58–3.10	<0.001
yes	69.09	409	30.91	183	7.80	5.59–10.90	<0.001
definitely yes	88.96	137	11.04	17	28.14	15.77–50.20	<0.001

a Non-response was observed in 24 cases out of 1560.

### Reasons for COVID-19 vaccination hesitancy

**Figure 1:**
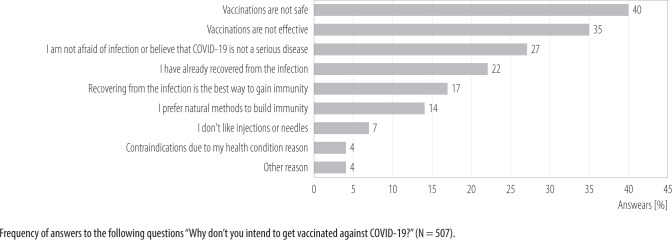
Reasons for COVID-19 vaccination hesitancy among a nationally representative sample, Poland, October 7 – November 28, 2021

Among the reasons for not intending to get vaccinated against COVID-19, respondents most often indicated that vaccinations are not safe (40%) and not effective (35%), followed by the belief that they are not afraid of infection or that COVID-19 is not a serious disease (27%), or that they have already recovered from the infection (22%). Additionally, some people believe that recovering from the infection is the best way to gain immunity (17%) or use natural methods to build immunity (14%). Fear of injections or needles was declared by 7% of respondents, and 4% indicated contraindications due to their health condition (Figure 1).

### Attitudes towards COVID-19 vaccination

The decision to get vaccinated against COVID-19 is correlated with attitudes towards vaccinations and perception of the pandemic. Positive correlation was found with 9 statements and negative correlation with 12 statements. The strongest positive correlations were with the following 3 statements:

– “A person who gets vaccinated protects not only themselves but also others” (r_s_ = 0.59142),
– “COVID-19 vaccines are a success of modern medicine” (r_s_ = 0.51348),
– “Medical experts appearing in the media encouraging vaccinations are credible to me” (r_s_ = 0.44798).

The strongest negative correlations were with the following 5 statements:

– “COVID-19 vaccines may contain dangerous substances or other elements” (r_s_ = –0.53515),
– “Mass vaccination of the population against COVID-19 is a medical experiment that should not be allowed” (r_s_ = –0.54769),
– “COVID-19 vaccines cause genetic changes and lead to mutations” (r_s_ = –0.50838),
– “The media exaggerate the threat of the coronavirus' infections” (r_s_ = –0.49299),
– “Patients do not die from COVID-19 but from comorbidities” (r_s_ = –0.46577).

The correlation coefficients for all variables studied are presented in Table 4. The results are statistically significant (p < 0.001).

**Table 4:** Spearman's rank correlation coefficient between COVID-19 vaccination and individual vaccination-related statements among a nationally representative sample, Poland, October 7 – November 28, 2021

Statement	r_s_^a^
A person who gets vaccinated protects not only themselves but also others	0.59142
COVID-19 vaccines are a success of modern medicine	0.51348
Medical experts appearing in the media encouraging vaccinations are credible to me	0.44798
Vaccinating is the most effective way to protect children from serious diseases	0.39429
Vaccines for children are safe	0.39323
Overall, vaccinating children does more good than harm	0.37200
Parents of vaccinated children are adequately informed about the side effects of vaccines	0.35867
Thanks to vaccinations, many serious diseases in children are now virtually non-existent	0.33143
Getting vaccinated is in accordance with my religious beliefs	0.28031
The SARS-CoV-2 virus was created by humans	–0.24137
Instead of vaccination, it is better to let a child go through a contagious disease, because it's nothing to be afraid of – a few days with measles, chickenpox, or rubella and then we have natural immunity for life	–0.26015
In the first years of life, children receive too many vaccines	–0.28541
Vaccines for children can cause serious side effects and complications	–0.29368
Vaccines for children can cause serious developmental disorders such as autism	–0.33788
Vaccinations are promoted not because they are genuinely necessary, but because it is in the interest of pharmaceutical companies	–0.35098
Vaccinations weaken child's natural immunity	–0.30207
Patients do not die from COVID-19 but from comorbidities	–0.46577
The media exaggerate the threat of the coronavirus' infections	–0.49299
COVID-19 vaccines cause genetic changes and lead to mutations	–0.50838
Mass vaccination of the population against COVID-19 is a medical experiment that should not be allowed	–0.54769
COVID-19 vaccines may contain dangerous substances or other elements	–0.53515

a The results range from 1 to –1, where: 1 indicates strong support for the statement, and –1 indicates strong negation of the statement.

## DISCUSSION

The conducted analysis illustrates how attitudes and opinions of young Poles towards vaccinations influence health decisions (decision to accept the COVID-19 vaccine). The study conducted during the COVID-19 pandemic (a real health threat) may serve as a crucial element in forecasting future societal behaviors towards new health challenges public education efforts should be implemented via channels that young people find credible and accessible – such as verified social media campaigns, influencers with medical or public health credentials, independent NGOs, or school-based educational programs.

Merely increasing factual knowledge is insufficient if the message is delivered by institutions perceived as untrustworthy. Restoring credibility to public health authorities and improving communication strategies should therefore be treated as a parallel priority.

### Justification for the selection of the group

The target group was selected to include individuals who currently or in the near future will make decisions regarding vaccinations for their own children. Given that the last vaccination in the National Immunization Program is administered at age 19 years, it was assumed that some respondents had recent experiences with their own vaccinations. Additionally, preliminary information about the progress of the COVID-19 vaccination program indicated that the 15–39 years age group had significantly lower vaccination rates compared to older age groups, making their analysis valuable for understanding vaccination decision-making. At the time of the study, COVID-19 vaccinations for individuals aged ≥15 had been freely available for several months.

### COVID-19 vaccination status

In the surveyed population, during the study, the percentage of people vaccinated against COVID-19 was 52%. According to Our World in Data [4], the average vaccination rate in Poland across all age groups was just <60%. The average for the entire European Union is 73%, indicating that Poland has one of the lowest vaccination rates.

### Factors related with COVID-19 vaccination and COVID-19 vaccination hesitancy

Many studies have examined factors associated with vaccination acceptance and hesitancy [5,6], but few studies have examined determinants influencing the decision to vaccinate against COVID-19 in the general population of young Poles. The most analyzed factors were age, gender, education status and place of residence. Similarly to these results, some studies show that young adults' willingness to vaccinate increases with age [7,8], while others suggest the opposite [9]. There is agreement that vaccination acceptance is higher among individuals living outside rural areas and among those with higher educational and social status [10]. Some studies suggest that women are more reluctant to be vaccinated [11], while others suggest that men are more hesitant [12]. Hesitancy is higher among those without a partner [13].

COVID-19 vaccine acceptance and hesitancy depend not only on the economic and sociodemographic factors. Vaccine acceptance is associated with belief in the importance of collectivity, solidarity, and altruism [14]. It is also related to the degree of vaccine effectiveness and the incidence of side effects [15]. It is positively correlated with trust in modern medicine, scientific knowledge, and medical personnel, and negatively correlated with trust in homoeopathy and horoscopes, and the belief that one's immune system is sufficient to fight disease COVID-19 [16].

Exposure to misinformation about vaccination and conspiracy theories linked to COVID-19 related anxieties, as well as concerns about vaccine safety and potential side effects, influence hesitancy to vaccinate [17–19].

Political views and level of religiosity may also affect vaccine acceptance [20]. Voters of right-wing political parties are more likely to avoid getting vaccinated against COVID-19 than voters of other political parties [21]. Cancer patients, people who have not been vaccinated against influenza, and pregnant women are more reluctant to be vaccinated against COVID-19 [22,23].

Numerous studies indicate that vaccine knowledge is a crucial aspect of health literacy and a key factor in reducing vaccine hesitancy. Such a review indicates a relationship between having this knowledge and vaccination. This applies equally to vaccine hesitancy, attitudes towards vaccination, intention to vaccinate, as well as actual uptake of these vaccinations [24].

The analyses of the reasons for refusing the COVID-19 vaccine revealed that the underlying factors can be divided into 2 groups: modifiable and non-modifiable. This division assumes that it is possible to intervene to modify some factors, while others are beyond our control.

The modifiable factors included the following beliefs: “COVID-19 vaccines are not safe,” “I am not afraid of COVID-19 infection,” “COVID-19 is not a serious disease,” “I have already recovered from COVID-19, and I believe that the best way to acquire immunity is through infection.” Educational efforts make it possible to convince the public that vaccines are generally safe and that most symptoms appearing immediately after vaccination are not adverse effects of the vaccine but rather signs of the body developing immunity. Expanding public knowledge about the dangers posed by infection with a harmful virus or bacteria, not only to the infected individual but also to their loved ones and surroundings, will help mitigate the trivialization of contact with an infectious disease. Similarly, understanding the mechanisms of acquiring specific immunity through vaccination vs. natural infection will clarify that vaccines provide broad-spectrum immunity against various serotypes of bacteria or viruses. In contrast, contracting the disease not only risks complications or death but also provides limited immunity, protecting only against that specific pathogen.

Non-modifiable factors are considered to be those dependent on individual variables, such as natural preferences (“I prefer natural remedies/formulas to build immunity”), fears and anxieties about painful procedures (“I dislike injections and needles”), firmly established beliefs (“COVID-19 vaccines are not effective”), or cases where the patient has objective contraindications to receiving specific vaccinations.

While it is relatively straightforward to explain the link between a negative decision regarding vaccination and non-modifiable factors, the situation is more complex with modifiable factors. Implementing actions to change the perception of certain aspects related to infectious diseases and their prevention could significantly contribute to improving overall vaccination rates in the population.

Since the World Health Organization (WHO) identified vaccine hesitancy as 1 of the 10 greatest global health threats in 2019 [25], efforts aimed at improving vaccination rates worldwide against infectious diseases might be more effective if they focus on modifiable factors. Addressing these factors directly could significantly influence the decision-making process regarding vaccination.

Many studies indicate that maintaining or improving a person's quality of life throughout their lifetime depends on a high level of so-called health literacy. This is defined as the knowledge, motivation, and ability to find, understand, evaluate, and apply relevant health information in specific circumstances concerning oneself [26].

Therefore, improving and intensifying education on infectious diseases, the associated dangers, complications from past infections, the availability of preventive measures such as vaccines, and the safety of their use should be a priority in efforts to raise vaccination levels against various diseases globally. Generally, these efforts were termed “vaccine literacy” by the WHO in 2011. This concept, introduced by Ratzan [27], states that vaccine literacy is the ability of people to access, process, and understand basic knowledge about vaccines and immunization, as well as to evaluate the potential consequences and risks of their behavior and to make informed health decisions.

On the other hand, the results of the National Health Test of Poles conducted in 2021 and 2022 indicated a consistent percentage of Poles (13%) declaring that they would never get vaccinated against COVID-19 [28,29]. The main reasons cited by respondents for refusing vaccination were concerns about the safety of the vaccine (43% in 2021, 33% in 2022) and the occurrence of side effects (37% in 2021, 36% in 2022). Additionally, in 2022, 20% of individuals declaring that they would never get vaccinated cited their opposition to vaccinations as the reason, representing a 7-percentage point increase from 2021. The results of this study highlight a shift in the motivation for not getting vaccinated and may suggest that concerns are merely a pretext since, despite the decrease in concerns about vaccine safety, the refusal rates have not declined. Therefore, educational efforts can only be effective if they are also oriented toward “immunizing” the young Polish population against medical misinformation circulating in the public sphere. At the same time, it is essential to consider the multidimensional nature of Poles' attitudes towards vaccinations. The analysis that divided the Polish population into 6 homogeneous groups regarding attitudes towards vaccinations clearly indicates that communication efforts should be tailored to the needs of specific target groups. Reaching individuals with ambiguous attitudes is crucial for building and maintaining herd immunity [30].

### Limitations

The most important limitation in this study is that the results are based on declarations of the respondents, which may differ from their actual opinions or actions. The declarations regarding COVID-19 vaccination status of the respondents were not verified in any way. Despite these limitations, the present study offers valuable insights into the future vaccination attitudes of young Poles.

## CONCLUSIONS

The COVID-19 pandemic has shown that despite civilizational development and medical advances, there is still a real threat from infectious diseases. However, it has also become a topic used to polarize society and spread medical misinformation. As a result, the vaccination rate among young Poles is one of the lowest in Europe. Anti-vaccination proponents exploit fears about vaccine safety, which demonstrates that education alone is not enough. It is crucial to simultaneously improve the content and the credibility of the messenger. Communication must be tailored, trust-oriented, and delivered through media and platforms recognized as legitimate by the target group. The findings suggest that, unless trust-building and tailored communication efforts are undertaken, we may expect a continued polarization of attitudes among young Poles – with a stable subgroup resistant to vaccination, and a hesitant group whose decisions can be positively influenced by targeted interventions.
